# TRADITIONAL AND NONTRADITIONAL FAMILY DEMENTIA CAREGIVING AMONG SEXUAL AND GENDER MINORITIES

**DOI:** 10.1093/geroni/igae098.1320

**Published:** 2024-12-31

**Authors:** Jason Flatt, Krystal Kittle, Andrew Pickett, Maritza Dowling, Joel Anderson

**Affiliations:** University of Nevada Las Vegas, Las Vegas, Nevada, United States; University of Massachusetts Amherst, Springfield, Massachusetts, United States; Indiana University, Bloomington, Bloomington, Indiana, United States; George Washington University, Washington, District of Columbia, United States; University of Tennessee-Knoxville, Knoxville, Tennessee, United States

## Abstract

Few studies on dementia caregiving have examined the unique needs of sexual and gender minority dementia caregivers (SGM-DCs) who self-identify as lesbian, gay, bisexual, transgender, queer, intersex, asexual, and/or additional identities. The Alzheimer’s Association estimates that over 350,000 SGM older adults in the U.S. are currently living with dementia, a number projected to increase to over a million by 2050. At least 1 in 10 SGM caregivers are providing care to someone with dementia. This paper synthesizes findings about traditional (i.e., biological) and non-traditional (i.e., chosen) family structures from several studies led by our research team examining the health of SGM-DCs. We found 10-20% of SGM-DCs report caring for non-traditional (i.e., chosen) family members with dementia. We also found over half of SGM-DCs report not living with their care recipient. Using population-based data, we found a larger number of SGM-DCs who report caring for a grandparent with dementia compared with non-SGM–DCs (17% vs. 4%, respectively). SGM-DCs of chosen family members were more likely to report providing care for fewer than five years and fewer than 8 hours per week. Our analyses did not find significant differences in physical or mental health outcomes between traditional and non-traditional SGM-DCs. We highlight areas for future research exploring divergent support needs between traditional and non-traditional family caregivers, as well as related implications for health policy and intervention efforts to promote the health of SGM-DCs. Historical changes in dementia caregiving related to changes in family structure should also be examined in future research.

